# Cluster Randomized Controlled Trial to Promote Physical Activity Among Low-Resourced Mothers in New York City: Protocol for the Free Time for Wellness Effectiveness Trial

**DOI:** 10.2196/71381

**Published:** 2026-01-13

**Authors:** Jessica Watterson, Kate Magsamen-Conrad, Kajal Gokal, Ingrid Oakley-Girvan, Jennifer S Hirsch, Min Qian, Richard Buchsbaum, Charlene Niles, Kendra Van Horn, Stephanie Moshier, Andreina Martinez, Avery Garcia Flores, Sherece Laine, Marilisa Vega, Lauren C Houghton

**Affiliations:** 1 Institute for Health Transformation, School of Health and Social Development Deakin University Geelong Australia; 2 Department of Communication Studies University of Iowa Iowa City, IA United States; 3 School of Sport, Exercise and Health Loughborough University Loughborough United Kingdom; 4 Public Health Institute Oakland, CA United States; 5 Department of Epidemiology Mailman School of Public Health Columbia University New York, NY United States; 6 Jovie New York, NY United States; 7 New York City Department of Parks and Recreation New York, NY United States; 8 West Side Campaign Against Hunger New York, NY United States; 9 Herbert Irving Comprehensive Cancer Center Columbia University New York, NY United States

**Keywords:** public health, physical activity, low-resourced, mothers, chronic disease epidemiology, low income, chronic disease, childcare

## Abstract

**Background:**

Physical inactivity is pervasive and prevalent in the United States, particularly among women of low socioeconomic position and women with children. Structural and social barriers make active leisure time a rare commodity, creating a pressing health issue because physical inactivity increases the risk of chronic diseases and poor health.

**Objective:**

The broad objective of this study is to test the effectiveness of Free Time for Wellness, a multilevel intervention to increase physical activity among low-resourced mothers.

**Methods:**

This study comprises a 3-arm parallel cluster randomized controlled trial (RCT) with low-resourced mothers living in New York City. We will randomize fitness class sites (clusters) into arm A (contact control), receipt of free weekly fitness classes; arm B, receipt of free childcare combined with free weekly fitness classes; and arm C, receipt of free childcare combined with free weekly fitness classes and peer support activities. Over 2 years, we will recruit a pilot wave followed by 7 additional waves, totaling 720 participants into 24 fitness classes. Physical activity is the primary outcome, measured using accelerometers, but secondary outcomes also include physical activity assessed using a self-reported questionnaire and attendance data. We will assess additional secondary outcomes (eg, health status, depression, and anxiety) and mediators or moderators (eg, social support and cohesion) with a baseline and follow-up questionnaire. The intention-to-treat analysis will use linear mixed-effects models to assess the main intervention effects on physical activity outcomes and other secondary outcomes. Ethnographic methods will examine how intersecting forms of social identity shape women’s experiences of physical activity and understand how real-world conditions shape the intervention implementation.

**Results:**

The study received funding from the US National Institute of Health, covering the period of time from April 1, 2023, through March 31, 2028. We received initial institutional review board approval in August 2023. The study is active and recruiting participants. As of the day of manuscript submission, we have enrolled 471 participants. Data collection is anticipated to occur until September 2026 for primary completion. The estimated study completion date is December 2026. Dissemination of the results will take place with participants, community members, partners, and researchers through methods such as reports, websites, events, and academic publications and conferences.

**Conclusions:**

This cluster RCT tests whether access to childcare (an understudied structural barrier) and social support can increase physical activity. The study design and outcomes integrate ethnographic methods with a cluster RCT to better understand mechanisms and the impact of intersecting factors such as race or ethnicity, culture, gender, and socioeconomic position. The study leverages widely accessible, existing resources to promote physical activity and foster social support with the ultimate goal of assessing the effect of childcare access on parental health.

**Trial Registration:**

ClinicalTrials.gov NCT06654843; https://clinicaltrials.gov/study/NCT06654843

**International Registered Report Identifier (IRRID):**

DERR1-10.2196/71381

## Introduction

More than 46% of US men and women do not achieve the recommended physical activity guidelines for aerobic activity each week, and 76% do not meet the guidelines for both aerobic and muscle-strengthening activity [1]. This is important because physical inactivity contributes to an increased risk of chronic diseases and poor health. Physical inactivity is particularly prevalent in women of low socioeconomic position (SEP; 60% are inactive), suggesting that there are structural barriers to being physically active. Proposed mechanisms through which low SEP may influence physical inactivity include increased biological stress, increased risk of poor health due to reduced access to health care, and reduced access to other resources such as physical activity facilities and gymnasium memberships [2].

Our previous research identified that an additional barrier for women who are mothers is having the free time to be physically active [3]. This is also supported by other research showing that women with children tend to be less physically active than women of the same age without children, due to structural and social barriers that reduce active leisure time [4,5]. For resource-limited mothers in particular, social and environmental barriers to physical activity include family expectations, lack of transportation, insufficient financial resources, unsafe environments, and a lack of childcare [4]. Childcare and household duties cause significant stress and exhaustion for mothers, with mothers describing parenting as “relentless” (yet also acknowledging its rewards) [6]. There is a large existing body of literature examining wellness interventions for mothers. However, most studies focus only on promoting physical activity during pregnancy or the postpartum period (and not later in motherhood), when being physically active without childcare is easier because young babies can be included in the mother’s activities, such as going for stroller walks. This focus is illustrated by the large number of systematic reviews focused on interventions in these periods [6]. In addition, while a lack of childcare is a well-known barrier to mothers’ physical activity, backed by research, very few studies explicitly address this barrier as part of an intervention [4,7] For example, in a systematic review of group-based physical activity interventions for postpartum women, none of the studies provided childcare, although many studies have identified it as a barrier to being physically active [8].

This study will add to a small but important body of literature on how mothers’ health can be improved by addressing the unique challenges that mothers face in finding time and space for their health and well-being. Some limited evidence suggests that physical activity opportunities for mothers, when children are being cared for by others, can lead to reduced stress and improved BMI, lower resting heart rate, and normal blood pressure in comparison to controls [7,9]. An older systematic review of physical activity and parenthood found that “social support and childcare were paramount in alleviating physical activity barriers, especially for mothers” [8].

To address the need for mothers to find time and space for physical activity, we will test a first-of-its-kind intervention that we co-designed [3] and piloted with mothers [10], which provides free childcare in combination with a novel peer support system. Based on prior theory and our pilot work [11,12], we hypothesize that free childcare and peer support will increase physical activity, and no previous health interventions have studied this. Expanding research on social cohesion suggests that individual adoption of health behavior is much more likely when participants receive social reinforcement from multiple neighbors in their social network [13,14]. For example, a systematic review of randomized controlled trials (RCTs) of group-based physical activity for mothers of young children found evidence of increases to self-reported physical activity and psychological well-being [15]. Further, while resource-limited mothers reported that peer support encourages their participation in physical activity [5], another study found that higher-income mothers were significantly more likely to report friends or family offering to be physically active with them [6]. Our co-designed intervention, the Free Time for Wellness (FT4W) program, will generate novel access to individual- and community-level resources and will leverage peer support among mothers in urban, racially segregated, low-income communities in New York City.

The broad objective of this study is to test the effectiveness of FT4W, an innovative multilevel physical activity intervention to increase physical activity and ultimately reduce the risk of chronic diseases and improve mothers’ well-being. We will do so via the following specific aims:

- Aim 1: conduct a parallel cluster RCT to evaluate the effectiveness of providing free childcare at free fitness classes to increase physical activity among low-resourced, historically minoritized mothers.
- Aim 2: test if peer support enhances the effect of the childcare intervention on physical activity by including another intervention arm promoting social cohesion.
- Aim 3: integrate ethnographic methods throughout the RCT to understand how individual-, community-, and institutional-level factors modify and influence the effect of the intervention, with particular attention to intersectional inequalities.

The saying “it takes a village” captures an existing cultural phenomenon of mothers supporting mothers through in-person and virtual “mom groups” [12]. FT4W builds from this concept, combining cultural context, the science of social cohesion, co-design methodology, and mixed methods to bolster mothers’ support networks and help promote their wellness. If effective, the FT4W program will illustrate how supporting mothers at the community level can increase physical activity, thus reducing chronic disease disparities in communities with high social inequality by providing resources, building social cohesion, and freeing up time for mothers to focus on their own wellness.

## Methods

### Study Design

This study comprises a 3-arm parallel cluster RCT with low-resourced mothers living in New York City. The trial has been planned, and this protocol is reported in line with the CONSORT (Consolidated Standards of Reporting Trials) statement [16].

The complete FT4W program includes free childcare at weekly fitness classes and peer support activities. We will randomize fitness class sites (clusters) into arm A, a contact control, receiving only the free weekly fitness classes; arm B, receiving free childcare provided at the same location as free weekly fitness classes; or arm C, receiving free childcare at free weekly fitness classes, plus peer support activities.

### Theoretical Framework and Logic Model

The FT4W program is founded on social cognitive theory, which posits that human behavior is the product of the dynamic interplay of personal, behavioral, and environmental influences [17]. Social cognitive theory emphasizes reciprocal determinism in interactions between people and their environments. The majority of physical activity interventions use social cognitive theory as indicated by the theory and technique tool developed by the Human Behaviour Change Project [18]. We used the theory and technique tool to align the FT4W program components and mediating pathways with behavior change techniques and mechanisms of action substantiated in behavior change literature and expert consensus. Studies that use behavior change techniques of restructuring the physical environment and peer support show that the mechanisms of action are environmental context and resources (aspects of a person’s situation or environment that discourage or encourage the behavior) and social influences (interpersonal processes that can cause oneself to change one’s thoughts, feelings, or behaviors) [18]. For our study, we will measure social influence via social cohesion.

**Figure 1:**
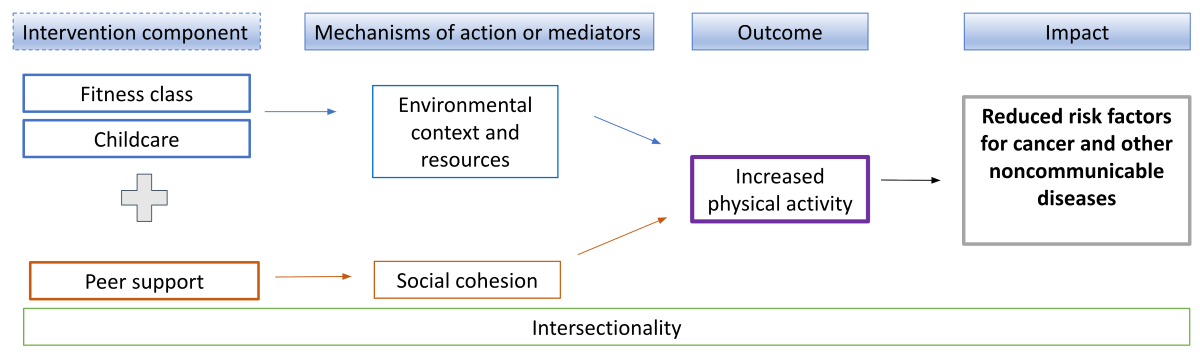
Logic model for Free Time for Wellness program.

Our logic model (Figure 1) illustrates the 2 complementary components of the FT4W program and mediating pathways in which the intervention can be effective. We hypothesize that providing free childcare during free fitness classes can provide environmental resources that participants can use to increase their physical activity. We hypothesize that this effect will be stronger in participants who also receive peer support, the intervention component that creates social cohesion. We also hypothesize that the effect of the intervention will differ based on race and ethnicity and other social stratifiers (migration status, SEP, and gender or sexual orientation).

### Patient and Public Involvement

The intervention was developed in this New York City–based study through a co-design process with women from Washington Heights, New York City [3,10]. We established collaborations with 3 community partner organizations (Shape Up NYC, Jovie, and the West Side Campaign Against Hunger [WSCAH]). These partners participate in research team meetings and in the study design. Partner details are provided as follows:

- Shape Up NYC: New York City Department of Parks and Recreation programs include Shape Up NYC, a free group fitness program offering 87 fitness classes at 60 locations across the 5 New York City boroughs. Shape Up NYC will determine and schedule available study sites and classes in coordination with the intervention.
- Jovie: Jovie is a professional childcare company that provides care for families and businesses. We partnered with Jovie during the feasibility study, and they will continue providing childcare.
- WSCAH: WSCAH is one of the largest food pantries that serves New York City and is distinctly committed to providing healthy food through a customer-choice supermarket-style pantry. WSCAH will coordinate volunteer activities and food delivery for participants in the arm receiving peer support.

These partners are members of our community advisory board (CAB). The CAB also includes 4 community liaisons and 2 community champions. The community liaisons are individuals who represent or serve our target communities, the community partners represent our organizational partners, and the community champions are local women who will help facilitate peer support activities.

The main CAB functions are to ensure that the study’s research strategies and design respect and serve our participants and make sense “in the real world.” The CAB will meet quarterly to troubleshoot any problems with the trial procedures. They will also bring community concerns or ideas to the research team.

### Participants

#### Eligibility Criteria

The trial will enroll mothers aged 18 years and older, who understand or speak English or Spanish, have children younger than 12 years of age (because younger children require more care, and we are focused on preventing chronic disease among premenopausal women), own a mobile phone, and live in nearby zip codes surrounding Shape Up NYC sites. Mothers must also meet income requirements, with total household income less than or equal to 165% of the area median income (calculated by household size).

#### Clusters

**Figure 2:**
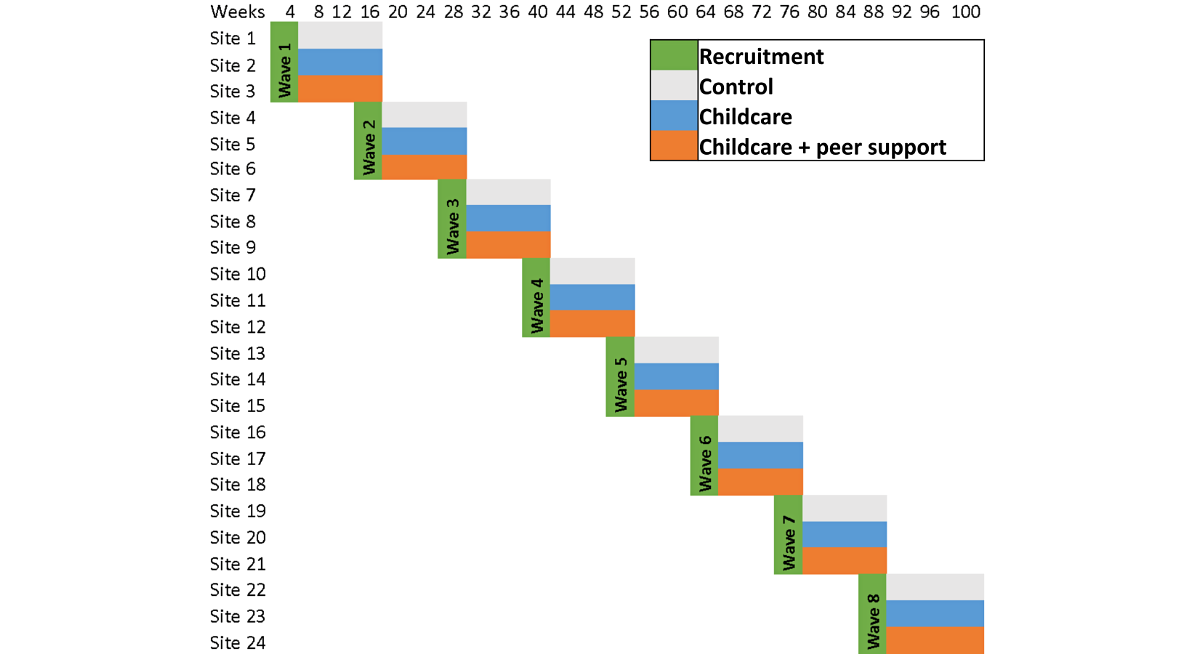
Staggered recruitment.

We will recruit 720 participants into 24 Shape Up NYC classes, across 1 pilot wave (n=90) and 7 study waves (n=630) over 2 years (Figure 2). Shape Up NYC class sites that allow research and have adjacent space for childcare will be eligible for selection. We will randomize each wave of 3 sites into 1 of 3 arms, using computer-generated random numbers, and run the program for 12 weeks. We will first randomize the class sites and then recruit participants into them while blinded.

For each wave, 30 participants will be recruited into each class (n=90 per wave). This process will be repeated for each wave with 3 classes, some of which will be located at the same sites as previous classes in previous waves. We will stratify randomization by geographic location and across seasons. While we cannot stratify on individual-level factors in a cluster RCT, stratification by geographic location will help to capture many social demographic factors at the individual level (eg, race or ethnicity and income level). In addition, Shape UP NYC trains fitness instructors at all sites following the same curriculum, and all arm C participants will have the same community champions (described more in the Peer Support Activities (Arm C Only) section).

#### Blinding

The biostatistician will randomize deidentified fitness class sites into arms and will keep this blinded from the principal investigator (PI: LCH) and project coordinators while recruitment occurs. All the study team members will be blinded except for the senior ethnographer, field ethnographer, community champions, and community partners.

#### Sample Size

We powered the study to compare the main intervention effects on the primary outcome, which is the average time spent in moderate to vigorous physical activity (MVPA) per week. We plan to enroll 7 sites per study arm (excluding the pilot wave), with about 30 participants with up to 45 children per site. We assume an attrition rate of 30% based on our feasibility study as well as another similar study [19] and an additional 25% accelerometer nonadherence rate based on previous studies [19,20], yielding an average 15 participants per site. Previous studies show that the intraclass correlation coefficient is ~0.15, and we expect a coefficient of variation of 0.5 in the number of participants across different sites. For aim 1, with 80% power, the minimum detectable effect size for the comparison of intervention arm B versus control arm A on average time spent in MVPA is Cohen *d*=0.68 at 5% significance. For aim 2, we will use the Bonferroni correction to adjust for multiple comparisons of arm C versus arm B and arm C versus arm A. With 80% power, the minimum detectable effect size for each comparison is Cohen *d*=0.75 at 5% significance. A previous physical activity intervention study with mothers led to an 80-minute increase in average time spent in MVPA [20]. Specifically, at posttest, the experimental group performed a mean of 154.8 (SD 45.3) minutes of physical activity, whereas the control group reported a mean of 74.6 (SD 44.4) minutes. This yields an effect size of *d*=1.8; thus, we are adequately powered for aims 1 and 2. Finally, quantitative analyses are not fully appropriate to examine intersectionality (eg, too reductionist), rendering a power analysis inappropriate; instead, we will integrate the statistical analysis with qualitative data to evaluate aim 3.

### Recruitment

We will use multiple recruitment methods to identify and recruit participants. We will distribute paper and digital flyers and advertise FT4W on social media platforms including Facebook and Instagram. WSCAH, Jovie, and their partner community-based organizations will circulate our recruitment flyer to their constituents via email. Our project coordinator will do direct in-person outreach at community events and schools. The project coordinators will follow up with individuals interested in participating in the study by phone, email, or SMS text message.

To mask the purpose of the study, we will tell potential participants that the study seeks “to find the best way to connect moms living in the same neighborhood.” Eligible participants will provide informed consent after reviewing the institutional review board–approved document that explains the study and their involvement in it by providing their digital signature.

### Interventions

#### Fitness Classes (All Arms)

Shape Up NYC offers classes that occur weekly for 1 hour free of charge to consenting participants in all 3 arms. Trained Shape Up NYC staff or volunteers lead the fitness classes, which include activities such as yoga, Zumba, and dance fitness. We will invite participants to attend the same scheduled class each week for the duration of their involvement in the 12-week study.

#### Childcare (Arms B and C)

Arms B and C will receive childcare from Jovie in an adjacent space to the fitness class.

#### Peer Support Activities (Arm C Only)

##### Overview

In addition to the fitness class and childcare, arm C will also receive peer support activities. Peer support activities will consist of group smartphone messaging facilitated by community champions, volunteer activities at food pantries, free deliveries of food pantry groceries, and group play dates at local playgrounds.

We structured group activities within the context of intergroup contact theory [21], social penetration theory [22], updated meta-analyses of this work [23,24], and research team members’ prior experience [25-27]. In short, positive communication and disclosure are essential to relationship building, which builds social cohesion. Sharing personal information (disclosure) and spending time together yield the largest effects in meta-analyses—these forms of intimacy grow over the course of developing relationships [22], and growth is more likely when anxiety is reduced and comfort is increased [28]. Anxiety reduction is the most important factor during the initial stages of developing social cohesion, hence our strategic structuring of peer support activities. Our intervention includes a task-based interaction (volunteer activities) where cognitive demand is low and interactional roles are clear. The focus is on the task, rather than developing relationships, reducing the pressure of interaction. As the volunteer activities continue, they also offer the opportunity for group members to enhance empathy, another salient component of cross-group relational development. Mediated interaction (group messaging) supports new relational development through a disinhibiting effect, lowering inhibitions and increasing comfort and disclosure [29].

##### Community Champions

Two bilingual (Spanish or English) community champions will foster social support and participation among arm C participants.

##### Weekly Playdates

Participants in arm C will be offered weekly group playdates for a total of 12 during the intervention. We will invite participants to bring their children to playgrounds in Shape Up NYC neighborhoods, where mothers can collectively watch their children play but also chat and reinforce social bonds. The first 2 playdates will have community champion–facilitated activities to help mothers get to know each other and to reduce the social effort and anxiety often associated with meeting new people.

##### Food Pantry Volunteering

We will invite arm C participants to volunteer as a group to help package food boxes during monthly events with WSCAH at one of their main locations. There is no childcare available for this activity, so participants will be told that they should not bring their children. Most volunteering opportunities are during the day (around 10 AM) on weekdays, and shifts are a maximum of 2-3 hours.

##### Free Grocery Delivery

In the first week of the intervention, arm C participants will also be provided with a link to WSCAH’s enrollment to opt into the home delivery of free groceries once per month for the 12-week intervention. There are no eligibility criteria to receive food from WSCAH, and participants will be eligible even if they do not volunteer.

#### Text Messages (All Arms)

##### Arms A and B

Arms A and B will receive individual text messages with a reminder for their weekly fitness class only.

##### Arm C

Arm C will be assigned to a messaging group with other mothers through WhatsApp, where community champions will “nudge” peer support and participation in activities through group messages. Community champions will follow scripted messages that remind participants when their next fitness class or peer support activity takes place. In addition, they will send weekly (prescripted) conversation prompts to “nudge” social interaction and will be able to “go off script” in response to organic group interactions. Participants will be allocated to groups first by language preference (ie, some groups may be in Spanish and some in English). The goals of the text messaging groups are (1) to encourage relationship-building, (2) to encourage participation in activities, and (3) to provide social support of various types (emotional, informational, etc).

### Outcomes

#### Primary Outcome: Physical Activity

The primary outcome will be total minutes of time spent in MVPA per week. We will objectively assess weekly MVPA, total physical activity, sedentary time, and sleep using a blinded research-grade wrist-worn accelerometer (Axivity AX3) in all groups.

#### Secondary Outcomes

##### Self-Reported Physical Activity

To overcome missing accelerometer data, we will gather self-reported physical activity data using the validated International Physical Activity Questionnaire (IPAQ) [30]. IPAQ asks participants to report the types and frequency of physical activities they have participated in over the past 7 days. IPAQ is reported as a score used to categorize people as having high, moderate, or low levels of physical activity.

##### Health Status and Well-Being

We will measure health status and well-being using 2 standardized surveys: EuroQol 5 Dimension 5 Level [31] and ICEpop Capability Measure for Adults [32]. EuroQol 5 Dimension 5 Level is a generic measure of health status that includes 5 dimensions: mobility, self-care, usual activities, pain, and anxiety or depression. ICEpop Capability Measure for Adults will capture the broader concept of well-being via 5 dimensions: feeling settled and secure; love, friendship, and support; being independent; achievement and progress; and enjoyment and pleasure.

##### Implementation Outcomes

###### Maintenance and Sustainability

Recent advancements in implementation science distinguish maintenance from sustainability by defining the amount of time, with maintenance lasting 6 months or more and sustainability beginning at 1 year or more. We will assess maintenance and sustainability at the organizational level through interviews with 6 stakeholders, 3 from Shape UP NYC and 1 each from Jovie, WSCAH, and the study team. We will use the Extension of the Reach, Effectiveness, Adoption, Implementation, and Maintenance framework to guide our questions.

###### Fidelity

The fidelity of the intervention will be measured during the 12-week trial periods in each wave. We will measure the dose delivered in each cluster by tracking the number of fitness classes and peer support activities offered, childcare availability, and the number and type of texts sent in each group. We will also measure the dose received according to attendance to all components of the intervention (fitness class, childcare, play dates, and volunteer activities) and the type and number of text message engagements. The field ethnographer will also observe how the fitness instructor delivers the class (physical activity level) and the interactions among participants before, during, and after the class (social cohesion).

###### Attendance

An additional process outcome will be attendance at fitness classes. Participation rates are arguably the most important process outcome of interest when evaluating the success of an intervention like FT4W. Shape Up NYC takes attendance at each fitness class, and community champions will take attendance for the peer support activities to understand participation, retention, and attrition levels.

##### Mechanism of Action (Effect Mediators) and Modifiers

###### Resources

We will ask participants to report resources they have access to at baseline and follow-up. We will ask the same FT4W-specific questions as in the feasibility study [10] and use the following constructs from the Social Determinants of Health Collection of the PhenX Toolkit, a set of standardized measurement protocols used in health equity research [33,34]: health insurance coverage and food insecurity. We will also measure access to childcare [35].

###### Social Cohesion and Support

We will measure participants’ perception of social cohesion in their neighborhood with 4 previously validated, standardized questions from the 2018 National Health Interview Survey using a 5-point Likert scale [36]. Questions ask about participants’ perceptions of help availability, neighbors’ accountability, trust in neighbors, and close-knit neighbors in their neighborhood. We will also administer the Medical Outcomes Study Social Support Survey, which asks participants to rate how often different types of social support are available to them when needed, such as someone to confide in or someone to help with chores if sick [37]. Finally, we will also administer the Sense of Community Index-2 at follow-up only (specifically asking about the FT4W group), which captures group belonging by examining the intersection of interpersonal and community influence in the sociocultural environment, byproducts of human relationships that are difficult to capture by measuring social networks and peer support. Previous evidence demonstrates that the Sense of Community Index-2 strongly predicts behaviors such as participation [38,39].

###### Intersecting Social Stratifiers

Intersectionality considers the interaction of different social stratifiers (eg, race or ethnicity, indigeneity, gender, class, sexuality, geography, age, disability or ability, migration status, and religion) and underlying multilevel power structures [33]. We will administer questionnaires at baseline and follow-up and use the responses for individual quantitative data. At baseline, we will assess individual social stratifiers using measures from the Social Determinants of Health Collection of the PhenX Toolkit [40]: ethnicity and race, biological sex assigned at birth, gender identity, sexual orientation, educational attainment (individual), health insurance coverage, and current employment status. We will also ask about migration status, language preference, number and age of children, as well as the degree to which their neighborhood supports physical activity through the Physical Activity Neighborhood Environment Survey [41]. While previous health disparity research considers forms of social stratification in isolation or in an additive manner [40], intersectionality is multilevel and calls for analysis that is more than just a sum of its parts. Thus, quantitative methods are limited. We will use qualitative methods to explore how intersecting factors at the individual, community, and institutional levels modify effectiveness and implementation. We will specifically explore how SEP, race, and ethnicity intersect when it comes to the FT4W experience.

#### Data Collection

##### Quantitative Data Collection

###### Baseline Assessment

Participants will receive individualized links via text message to complete the online baseline questionnaire, which assesses participant demographics, physical activity, health status and well-being, social cohesion, access to childcare, and individual- and community-level social determinants of health.

Study staff will send a preprogrammed Axivity AX3 watch mailed directly to the participant following completion of the baseline survey (week 0). Participants will be asked to wear the Axivity watch on their nondominant wrist for 24 hours per day for 8 full days and to complete the accompanying sleep diary every day, either on paper or via text message, based on their preference. Instructions on how to use the watch and complete the diary will be included in the parcel via written materials and a brief video. Following 8 days of wear, the participant will post the accelerometer (free of charge in prestamped envelopes) back to the project coordinator, who will check the data for completeness. If the data do not meet our quality control checks and there are at least 2 weeks remaining before the first scheduled class, then we will mail another accelerometer to the participant. A previous study conducted with the same population found nonadherence to waist accelerometers to be 35% [42]. Data from the National Health and Nutrition Examination Survey also show that adherence to wrist accelerometers is higher (70%-80%) than waist accelerometers (40%-70%); therefore, we expect adherence to wrist accelerometers to be 75% in our study [43]. To increase adherence, we will send automated text reminders to confirm receipt of the accelerometer, to complete the accompanying sleep diary every day, either on paper or via text message, and to return the accelerometer.

###### Follow-Up Assessment

At week 10, study staff will again mail Axivity AX3 watches to participants and links to complete the online follow-up questionnaire, following the same procedure as baseline. We will consider them lost to follow-up if we do not receive a response by week 16.

##### Qualitative Data Collection

###### Overview

Ethnographic methods will be used to examine how intersecting forms of social inequality shape women’s experiences of physical activity in the context of their broader lives (studying the experiences of research participants) and to understand how real-world conditions shape the intervention implementation (studying the workings of the research team and the impacts of the changing social context on study implementation). The field ethnographer will be bilingual and of the same approximate age as the research participants and will be supervised by the team’s senior ethnographer.

###### Ethnographic Research With Study Participants and Intervention Activities

The field ethnographer will conduct participant observation, which will entail participating in at least 6 fitness classes (2 in each arm) and 4 peer support activities in each study wave throughout the trial. Through participant observation, the ethnographer will use a structured note-taking form to record observations about group dynamics, social environment, expressions of cultural norms, expressions of frustration at challenges of getting to exercise class, and other individual comments or social interactions relevant to understanding challenges women with children face in engaging in physical activity, including related to childcare, as well as how the intervention assets (childcare and social support) do or do not facilitate their participation in Shape Up NYC classes. For each instance of participant observation, the ethnographer will also record the time of day, weather, and time of year to assess temporal impacts on participation. The ethnographer will take both brief field notes on their phone during childcare drop-off, before and after classes, and during social support activities, expanding these into more complete notes after leaving the study site. Notes will describe the physical conditions, the effect of mother-child dyads at drop-off and pickup, how participants engage with each other and the instructor, and what is said or expressed through body language about the classes and activities.

Group message data for arm C will serve as an additional source of qualitative data. Through content analysis, researchers will assess topics of discussion, jokes, expressions of friendliness, the development of connections between research participants, the sharing of information, and other content types to shed light on what social support consists of and how it might affect participation in physical activity. We will also quantify the number of interactions in the chat per wave.

###### Ethnographic Case-Control Research

Through comparing participants who engage in high and low rates of physical activity, ethnographic case-control research may shed light on otherwise unmeasured factors that affect the outcome as well as social, cultural, and structural barriers to physical activity. Purposive sampling for the ethnographic case-control study will use data from accelerometers to identify women with low and high levels of each outcome and then recruit individuals who meet the criteria for diversity specified in Table 1. We will select pairs of cases and controls within arms and match on race and ethnicity. We will select pairs from all arms within the same wave.

**Table 1:** Ethnographic case-control sample.

Race and ethnicity			High physical activity, n		Low physical activity, n
**Black**					
	Non-Hispanic	4		4	
	Hispanic	4		4	
**White**					
	Hispanic	4		4	

After the week 12 follow-up, the field ethnographer will conduct semistructured interviews with those recruited for this purposive sample to gain further insight into participants’ views and experiences with FT4W and specifically to understand differences in individual characteristics, social context, or access to resources, which might lead the intervention to be impactful for some and not for others. Interviews will be audio-recorded and transcribed. We expect to complete ~48 interviews or until we reach data saturation as recommended for this type of study [44].

###### Development and Fidelity of Intervention Protocol

Ethnographic research conducted by the senior ethnographer, with a focus on the development of the intervention itself, will generate an understanding of how factors internal to the team (team process, particularities, and power dynamics) and factors external to the team (the political and social context) shape the development and implementation of the intervention protocol, including fidelity to the proposed intervention.

The study itself is an object of inquiry. To do this, the senior ethnographer will participate in all study team and CAB meetings and use a checklist to track development, fidelity, and changes to the protocol based on real-world demands. In addition, the senior ethnographer will conduct key informant interviews with study team members and community partners to learn about community organizations’ experience of the intervention implementation, including policy factors and other potential challenges to sustainability. Finally, over the duration of the project, the senior and field ethnographers will compile information about how the broader social context (eg, budget cuts due to the migrant crisis in New York City) shapes the implementation of the project.

#### Data Monitoring

Data monitoring by the study team will occur at least on a biannual basis (ie, every 6 months). Participant enrollment (including compliance with protocol enrollment criteria), data completeness, and the status of all enrolled participants will be reviewed by the PI and supported by the study team.

This study will be stopped prior to its completion if (1) the intervention is associated with adverse effects that call into question the safety of the intervention, (2) difficulty in study recruitment or retention will significantly impact the ability to evaluate the study end points, (3) any new information becomes available during the trial that necessitates stopping the trial, or (4) other situations occur that might warrant stopping the trial.

#### Analyses

##### Quantitative Analysis

The primary analyses will be on the intention-to-treat sample: all randomized participants according to the assigned treatment. Missing data on outcome variables will be dealt with by using multiple imputation or inverse probability weighting of cases with complete data, where we will calculate weights based on the probability of a participant being a completer versus a dropout. We will conduct a sensitivity analysis to provide a range of plausible effect estimates that could arise due to nonignorable missing data.

For aims 1 and 2, we will use linear mixed-effects models (LMMs) to assess the main intervention effects on physical activity outcomes and other secondary outcomes. The outcome measure of interest will be the dependent variable; the treatment arm (categorical variable with 3 categories) will be the predictor. Cluster-specific random intercept will account for intraclass correlation. We will further adjust the model for baseline covariates that are not balanced across treatment groups (if any). Comparison of treatment arms can be tested by forming contrasts of regression coefficients in the models.

We will conduct mediation analysis to test whether environmental resources and social cohesion, described under working mechanisms, mediate the intervention effects. For each mediation analysis, we will conduct 2 LMM analyses. The first model will use the outcome measure as a dependent variable and treatment arm and mediator as predictors. The second model will use the mediator as a dependent variable and treatment arm as the predictor. Both models will include a cluster-specific random intercept to account for intraclass correlation. Causal mediation effects will be tested using the R package *mediation*. For aim 3, moderator analysis will examine whether intervention effects differ across social stratification parameters (stratifier) at the individual and community levels. We will conduct a similar LMM analysis as in the main effect analysis, with the exception that each stratifier and its interaction with the treatment arm will be included in the LMM. The moderator effect will be examined by testing the stratifier by treatment interaction effect coefficients.

While 21 clusters are on par with the average cluster size in cluster RCTs, we still need to correct for the small sample since type I error can be up to ~7% for studies with 20-40 clusters [45]. For our continuous outcomes, residual (restricted) maximum likelihood will be used [46]. We will also adjust the test statistic to a 2-tailed *t* test with 2K–2 degrees of freedom if cluster structures are balanced [47]. If cluster sizes vary, alternative small sample corrections will be used. As a sensitivity analysis, cluster-level analysis will also be conducted. We will perform a 2-tailed *t* test on the cluster-level means of physical activity.

##### Qualitative Analysis

We will thematically code the ethnographers’ field notes and transcribe participant interviews for both deductive and inductive codes. A codebook will be created using thematic coding by reviewing field notes and transcripts, grouping information into meaningful categories, and creating descriptive labels. The ethnographer will apply the codes to all text. The PI will double-code 10% of the content. We will develop themes from the codes and assess where themes converge and diverge across the ethnographic case-control sample.

##### Quantitative and Qualitative Data Integration

**Figure 3:**
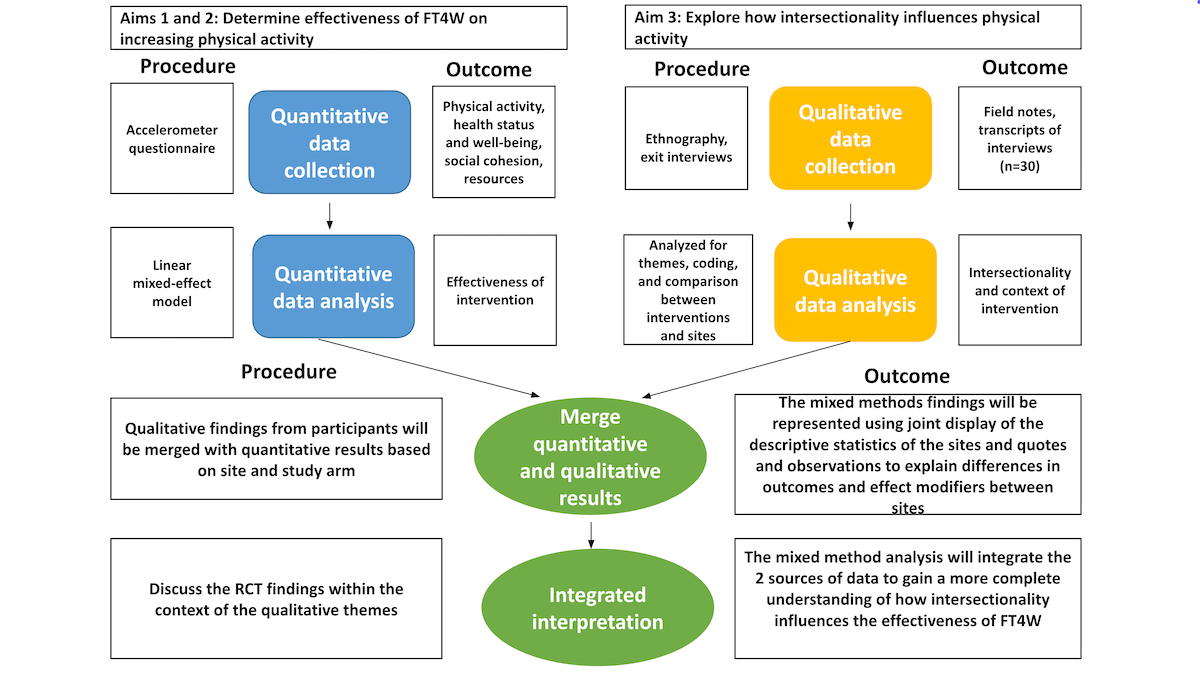
Embedded mixed method design. FT4W: Free Time for Wellness; RCT: randomized controlled trial.

Mixed methods analysis will integrate the cluster RCT results with the ethnographic data to better understand the underlying processes explaining why the intervention is or is not effective (Figure 3). Quantitatively, we will assess effectiveness and mediation. We will also test for additive interaction by stratifying the average effect from statistical models by race and ethnicity and income. However, because intersectionality is not additive, we will heavily rely on qualitative data analysis to understand how intersecting forms of social inequality shape participants’ physical activity, environmental resources, and social cohesion, all components that influence effectiveness as indicated in our logic model (Figure 1).

The quantitative and qualitative data will be integrated in several ways. First, we will examine where qualitative themes from the structured interviews converge and diverge based on accelerometer, social cohesion, and demographic data. We will use a joint display of cluster-level descriptive statistics and corresponding quotes to explain differences in outcomes and effect modifiers between clusters. Second, the participant observation of the intervention activities will reveal the unmeasured elements of the intervention that might affect either effectiveness or fidelity across clusters and arms. We will use narrative discussion to merge quantitative and qualitative data at the community level. Third, the institutional ethnography will provide preliminary implementation outcomes that we will use to develop future quantitative measures of fidelity, maintenance, and sustainability.

#### Timeline

Table 2 summarizes the schedule of enrollment, interventions, and assessments for 1 wave of study participants. As outlined in Figure 2, there will be a pilot wave followed by 7 waves.

**Table 2:** Schedule of enrollment, interventions, and assessments for 1 wave.

		Study period															
		Allocation	Enrollment	Postallocation													Closeout
			0	W1^a^	W2	W3	W4	W5	W6	W7	W8	W9	W10	W11	W12		
**Enrollment**																	
	Eligibility screen		✓														
	Informed consent		✓														
	Allocation	✓	✓														
**Interventions**																	
	Arm A (fitness class control)			✓	✓	✓	✓	✓	✓	✓	✓	✓	✓	✓	✓		
	Arm B (fitness class and childcare)			✓	✓	✓	✓	✓	✓	✓	✓	✓	✓	✓	✓		
	Arm C (fitness class, childcare, and peer support activities			✓	✓	✓	✓	✓	✓	✓	✓	✓	✓	✓	✓		
**Assessments**																	
	Baseline accelerometer (AX3) measurement		✓														
	Baseline questionnaire		✓														
	Attendance data collection			✓	✓	✓	✓	✓	✓	✓	✓	✓	✓	✓	✓		
	Follow-up accelerometer (AX3) measurement												✓	✓	✓		
	Follow-up questionnaire												✓	✓	✓		
	Interviews (for purposively selected participants only)															✓	

^a^W: wave.

#### Ethical Considerations

Columbia University’s institutional review board granted ethics approval (reference AAAU8303). All participants will provide written informed consent prior to participation, and the informed consent materials are provided in Multimedia Appendix 1. Only the PI, project coordinator, and the ethnographers will have access to identifiable data. All other research team members will have access to only deidentified data. Data will be stored on Columbia University computer systems in a custom-built database. Enrolled participants will receive a gift card after completing the baseline survey and returning the accelerometer and another gift card at week 12 follow-up after completing the survey and returning the accelerometer. Interviewees will receive another gift card in addition to the trial incentive.

## Results

The study received funding from the US National Institute of Health covering the period of time from April 1, 2023, through March 31, 2028. We received initial institutional review board approval in August 2023. The study is active and recruiting participants. As of the day of manuscript submission, we have enrolled 471 participants. Data collection is anticipated to occur until September 2026 for primary completion. The estimated study completion date is December 2026.

In line with our community-focused approach to research, we plan to communicate our study results back to the participants, partners, and New York City community using appropriate methods, such as reports, social media, or events. In addition, we will disseminate our approach and our findings to other researchers to help inform the further development and expansion of the FT4W intervention and similar interventions to promote physical activity among mothers. This will be done through the publication of this protocol, followed by reporting on any changes that take place during implementation, as well as the qualitative and quantitative results through academic publications and conferences.

## Discussion

### Anticipated Findings

The anticipated results from this study will increase our understanding of the potential roles of childcare and peer support in promoting physical activity among low-resourced mothers in New York City. The mixed methods results will also help us to better understand the mechanisms and the impact of intersecting factors such as race or ethnicity, culture, gender, and SEP on mothers’ physical activity. Finally, implementation results will help us to better understand how this program could be scaled up in the future, if successful, and how future similar programs should be designed.

### Strengths and Limitations

This study has several strengths. First, this cluster RCT tests a structural barrier to physical activity for mothers, lack of childcare, which is understudied. It also integrates ethnographic methods with a cluster RCT to better understand mechanisms and the impact of intersecting factors such as race or ethnicity, culture, gender, and SEP. Finally, the study leverages widely accessible, existing resources to promote physical activity (free Shape Up NYC fitness classes) and foster social support (WhatsApp groups to connect mothers living in the same area).

Despite these strengths, the study also has some limitations. First, it is limited to participants in New York City, which may limit its generalizability to other settings. The intervention includes options for Spanish-speaking participants, but not other languages, despite the diversity of languages spoken in New York City. This limitation, as well as the sample size, may result in an inability to capture a wide diversity of experiences. In addition, participants will only be followed for 12 weeks, so the study results will not be able to provide insight into potential long-term effects and sustainability. In addition, the use of accelerometers to collect physical activity data was not tested in our earlier feasibility study due to resource constraints. Finally, the use of existing Shape Up NYC fitness classes creates some potential for contamination across arms, but locations within a wave will be selected carefully to minimize this risk.

### Future Directions

The goal of the trial is to demonstrate that childcare and social cohesion are effective so that they can be implemented long-term in the future. If the intervention is effective, then the New York City local government will have data to justify financial support to implement the childcare component of their existing free fitness program. The implementation results from this study will help to identify what would be needed at the individual, implementation, and organizational levels for the intervention to be maintained and sustained over time.

### Conclusions

This cluster RCT tests a structural barrier to physical activity for mothers, lack of childcare, which is understudied. The study design and outcomes are geared toward integrating ethnographic methods with a cluster RCT to better understand mechanisms and the impact of intersecting factors such as race or ethnicity, culture, gender, and SEP. The study leverages widely accessible, existing resources to promote physical activity and foster social support with the ultimate goal of assessing the effect of childcare access on parental health.

## Appendix

Multimedia Appendix 1 Informed consent materials.

## Abbreviations

CAB: community advisory board
CONSORT: Consolidated Standards of Reporting Trials
FT4W: Free Time for Wellness
IPAQ: International Physical Activity Questionnaire
LMM: linear mixed-effects model
MVPA: moderate to vigorous physical activity
PI: principal investigator
RCT: randomized controlled trial
SEP: socioeconomic position
WSCAH: West Side Campaign Against Hunger
