# Supplementary material for: Cluster Randomized Controlled Trial to Promote Physical Activity Among Low-Resourced Mothers in New York City: Protocol for the Free Time for Wellness Effectiveness Trial
Source: JMIR Res Protoc. 2026 Jan 13;15:e71381. doi: 10.2196/71381 (PMC12848490; doi:10.2196/71381)
Supplement: Multimedia Appendix 1 [file resprot_v15i1e71381_app1.docx]

**Free Time for Wellness**

**Participant Information Sheet and Consent Form**

**Primary researcher**: Lauren Houghton

**Email**: lh2746@cumc.columbia.edu **Telephone**: 212-342-0246

**What is the project about?** This project aims to find out the best way to connect moms living in the same neighborhood. We would like to bring mothers living in the same neighborhood together for group activities, including fitness classes.

Because this is a research study, not everyone who participates in the study will be doing the same things. In this study, everyone gets free fitness classes. Some people will get fitness classes and free childcare. A third group will get free fitness classes with childcare and other activities (for example, group playdates). No one gets to choose which group they are in; we use a system that decides what group people are put in, this is called randomization.

**Who is the research funded by?** The project is funded by the National Institute of Minority Health and Disparities. The project team includes researchers from Columbia University, Loughborough University (UK), Public Health Institute, University of Iowa, University of Monash as well as community partners from Shape Up NYC, JOVIE and the Westside Campaign Against Hunger.

**Why have I been approached, and do I have to take part?** You are being invited to take part in the research study because you are a mom living in New York City. We are asking you to connect with other moms and join group activities. You can decide if you want to participate or not.

**What will happen in the study?** We are inviting you to participate in Free Time for Wellness study which includes participating in fitness classes provided by Shape UP NYC at no cost to you. We will send you a text message each week reminding you of the class. A member of our team will also observe some of the fitness classes, food pantry visits, and play dates.

Some participants will be a part of a group that receives free childcare. Childcare will be provided at the same location as the fitness class so that you can bring your children (who are <13 years) who will be cared for by a professional childcare service, while you participate in the fitness class.

For some participants, you may be included in a group chat with other parents attending the same fitness classes as you. For participants in this group, we will invite your group to monthly volunteer activities with a local food pantry. If you are in this group, you can have food delivered to your home. For these participants, we will also invite your group to weekly playdates at a nearby park. After the first survey, you may be put in contact with another mom (Community Champion) who will connect you to other moms in your neighborhood.

If you decide that you would like to take part, we will ask you to complete a survey and wear a activity watch called an accelerometer for 7 days at two different timepoints: now and in 12 weeks at the end of the study. Both surveys should take a 10-15 minutes to complete. After the 12 week follow-up period, we may contact you for a follow-up interview that will last about an hour. The interview will be recorded and transcribed and identifying information will be removed so the information can be analyzed by members of the research team. Once the analysis is complete, the recording will be destroyed and the transcript will be stored securely.

This study involves collecting the following personal information from participants: name, email address, gender, age and address. Your responses will be coded with a unique identifier instead of your name.

**What will happen to the information I give in the research study?** The information you give will be used to help the research team understand if the Free Time 4 Wellness program worked and provide the research team with guidance on how we could improve the resources to support other moms and families towards wellness. All personal data will be stored separately to survey data.

Identifiers will be removed from all materials and data and after such removal, we may use the data and information for future research studies. We may share some of the information you provide in written reports or presentations to other people who may be interested. However, all the information you provide will be anonymous and you will not be identifiable in these reports.

A description of this project will be available on http://www.ClinicalTrials.gov, as required by U.S. law. This website will not include information that can identify you. At most the Website will include a summary of results. You can search the Website at any time.

**Do I have to take part?** Your participation in the research is entirely voluntary, you don’t have to join. If you do decide to join the study but change your mind you are free to withdraw at any time without needing to give a reason and without any repercussions. Contact information can be found at the top of the page.

**Will my taking part in this study be kept confidential?** Yes. The research team at Columbia University will have access to your name and personal details from the questionnaires. If you are in the group that receives childcare, we will pass your name, your child’s name and child’s age to Jovie, the childcare provider. We will register your child into the NYC Parks Recreation Center where the fitness classes and childcare are provided. This means we will give NYC Parks your name as the emergency contact  along with your phone number and address and your child’s name and date of birth. You may sign waivers with our partners Shape Up NYC, Jovie, and West Side Campaign Against Hunger.  They will store that information for their record. We will use the waivers as a way of tracking attendance at the activities.

All the consent forms will be stored separately so your information will not be identifiable. The only time confidentiality would be broken is if we believed disclosing information could prevent harm to you or someone else.

The following people and/or agencies will be able to look at and copy your research records:

- The investigator, study staff and other professionals who may be evaluating the study;
- Authorities from Columbia University and New York-Presbyterian Hospital including the Institutional Review Board (‘IRB’). An IRB is a committee organized to protect the rights and welfare of people involved in research;
- The Office of Human Research Protections (‘OHRP’);
- The sponsor of this study, The National Institute of Minority and Health Disparities, including persons or organizations working with or owned by the sponsor may review your data for accuracy but may not copy information with your name on it.

This research is covered by a Certificate of Confidentiality from the National Institutes of Health. This means that the researchers cannot release or use information, documents, or samples that may identify you in any action or suit unless you say it is okay. They also cannot provide them as evidence unless you have agreed.  This protection includes federal, state, or local civil, criminal, administrative, legislative, or other proceedings. An example would be a court subpoena. 
   
There are some important things that you need to know.  The Certificate DOES NOT stop reporting that federal, state or local laws require. Some examples are laws that require reporting of child or elder abuse, some communicable diseases, and threats to harm yourself or others.  The Certificate CANNOT BE USED to stop a sponsoring United States federal or state government agency from checking records or evaluating programs. The Certificate DOES NOT stop disclosures required by the federal Food and Drug Administration (FDA).  The Certificate also DOES NOT prevent your information from being used for other research if allowed by federal regulations. 
   
Researchers may release information about you when you say it is okay. For example, you may give them permission to release information to insurers, medical providers or any other persons not connected with the research.  The Certificate of Confidentiality does not stop you from willingly releasing information about your involvement in this research. It also does not prevent you from having access to your own information.

**What are the advantages or disadvantages of taking part?** The advantages of taking part are to help us to understand if the new program works for moms living in the same neighborhood. There are no anticipated disadvantages to taking part in the questionnaires and surveys. However, if you feel uncomfortable with any of the questions then you have the right to withdraw at any point of the research without giving a reason and without any repercussions. Should you choose to participate in any of the physical activities or group activities offered through FT4W, please take extra care and stop if you feel you are no longer able to take part. There will not be any costs involved with taking part in the research.

**Compensation** You will receive up to $150 in gift cards. You will receive a $50 gift card after completing the first survey and wearing the accelerometer for 1 week. 12 weeks later, you will receive another $50 gift card after completing the second survey and wearing the accelerometer for another week. If also you participate in the interview, you will receive a third $50 gift card.

**What if there is a problem?** If there is a problem with the research or during any of the research activities, please speak to any member of the research team on the day or contact a member of the research team (contact details can be found at the top of the page.

If you have any questions about your rights as a research subject, you should contact the Columbia University Medical Center Institutional Review Board by phone at (212) 305-5883 or by email at irboffice@columbia.edu

**Statement of consent and signatures**

Statement of consent

I have read this consent form. The research study has been explained to me. I agree to be in the research study described above.

A copy of this consent form will be provided to me after I sign it.

By signing this consent, I have not given up any of the legal rights that I would have if I were not a participant in the study.

**Signatures**

________________________________________________________________

**Research Participant**  Date

___________________________________________________________________

Print Name of Research Participant

___________________________________________________________________

Individual Obtaining Consent Date

___________________________________________________________________

Print Name of Individual Obtaining Consent
